# More Positive Emotions During the COVID-19 Pandemic Are Associated With Better Resilience, Especially for Those Experiencing More Negative Emotions

**DOI:** 10.3389/fpsyg.2021.648112

**Published:** 2021-05-05

**Authors:** Jacob Israelashvili

**Affiliations:** Department of Psychology, The Hebrew University of Jerusalem, Jerusalem, Israel

**Keywords:** COVID-19, resilience (psychological), wellbeing, positive emotions, existential positive psychology (PP2.0)

## Abstract

The ongoing COVID-19 pandemic has taken a significant toll on mental health; people around the world are experiencing high levels of stress and deteriorated wellbeing. The past research shows that positive emotions can help people cultivate a resilient mindset; however, the reality created by the global crisis itself limits the opportunities for experiencing positive emotions. Thus, it is unclear to what extent their effect is strong enough to counter the psychological impact of the current pandemic. Here, the author reports the findings of a survey conducted across two large representative samples in the United Kingdom and the United States (*N*_total_ = 2000) during the first wave of the COVID-19 pandemic (in Spring 2020). A linear regression model revealed that the presence of positive emotions is strongly linked with resilience, in particular for individuals experiencing more negative emotions. These results show that positive emotions are particularly important to mental health in the context of high stress, reflected by increased levels of negative emotional experiences. These results are also consistent with the existential positive psychology perspective, which posits that even negative emotions can contribute to wellbeing once they are transformed. The author discusses the potential of positive emotions to transform suffering and thereby ameliorate the negative impact of the present collective crisis.

## Introduction

The COVID-19 pandemic has resulted in tremendous suffering, with more than 76 million people infected by the deadly virus (as of December 31, 2020). To mitigate the spread of the virus, imposed restrictions associated with poor psychological outcomes (e.g., reduced social contact; Wang et al., 2020) add further hardship to individuals and communities (Hwang et al., 2020). As a result, people are experiencing high levels of stress and deteriorated wellbeing, the magnitude of which has not been experienced since the last global pandemic in 1918.

Global pandemics are rare, but the stress-related ramifications introduced by the concrete or symbolic notion of death are common and reflect an inevitable part of life. Nothing can prevent individuals from facing difficulties, such as loneliness, death of a close other, loss of fertility, or deteriorating mental performance. Existential perspectives use this basic insight as a springboard to advocate that suffering is an inherent part of life that must be engaged, rather than avoided or fixed (e.g., Frankl, 1959; Van Tongeren and Showalter Van Tongeren, 2020; and see Positive Psychology 2.0; Wong, 2020). Pursuant to this is the idea that a person should be resilient; i.e., exhibit resourceful adaptability to stress and the ability to rebound (Block and Block, 2006). Accordingly, a resilient person will perceive life stressful experiences as “truths and not as threats” (Van Tongeren and Showalter Van Tongeren, 2020, p. 121).

The question is, what helps people to be positively engaged in perceiving situations as challenging, especially in times of prolonged exposure to extreme and new realities such as those imposed by the current global crisis? Several answers to this question appear in the scientific literature, such as the engagement in social activities (e.g., Killgore et al., 2020), reduced amount of daily hassles (e.g., Ahrens et al., 2021), and the lack of preexisting mental health problems (e.g., O’Connor et al., 2020). Another suggestion is that experiencing positive emotions, which elevate a good mood, can help people cultivate a resilient mindset (e.g., Sun et al., 2020). For example, studies on encounters with stress and coping show that preliminary positive emotions facilitate adaptive adjustment to stress, and assist in recovering from stressful events (Folkman, 1997; Lazarus, 2000; Fredrickson et al., 2003; Moskowitz et al., 2020). Similar findings were demonstrated by a meta-analysis of positive psychology interventions, which showed that elevating positive emotions enhances wellbeing and reduces depressive symptoms (mean *r* value estimated as 0.30; Sin and Lyubomirsky, 2009).

However, the applicability of such interventions in a time of global pandemic is questionable, as the global crisis itself limits the opportunities for experiencing positive emotions; for example, personal and communal activities (e.g., relationships and social gatherings for religious rituals) that help people to find meaning and overcome obstacles are forbidden or at least extremely limited. Moreover, the pandemic continues, already for 1 year, with no clear signs of ending in the near future (though recent new vaccinations give some hope). Hence, the question arises, whether positive emotions indeed have such a buffering effect in the context of the prolonged stress associated with the current pandemic. Furthermore, do positive emotions relate to better resilience among those who experience only them, or perhaps even for those who concurrently experience some level of negative emotions?

To answer these questions, we surveyed two large representative samples of participants in the United Kingdom and the United States during the first wave of the COVID-19 pandemic (Spring 2020). Participants were asked to report whether they have experienced a range of positive and negative emotions during the past week and answer questions measuring their level of resilience. We hypothesized that the level of positive emotions (only) would be positively linked to resilience, while the presence of negative emotions would be linked to deteriorated resilience. Furthermore, the study explored the possibility of an interaction effect, i.e., whether the relationship between positive emotions and resilience differs for those who experience different levels of negative emotions.

## Materials And Methods

### Participates

Two age, sex, and ethnically representative samples from the United Kingdom and the United States were recruited *via* Prolific Academic. *Sample 1* involved 1,000 UK participants ranging from 18 to 83 years old (*M*_age_ = 47, *SD*_age_ = 16; 52% female and 48% male). *Sample 2* included 1,000 US participants ranging from 17 to 83 years old (*M*_age_ = 46, *SD*_age_ = 16; 51% female and 49% male). A sensitivity analysis conducted in G-power suggested that with the standard criterion of *α* = 0.05, the regression analysis with three predictors (positive emotions, negative emotions, and their interaction) had a power of 0.80 to detect a small effect (*f*^2^ = 0.01). The Ethics Committee of the Psychology Department at the University of Amsterdam approved the study, and informed consent was obtained from all participants.

### Measures

#### Positive and Negative Emotions

Participants were asked to rate the emotional intensity of each one of twenty positive and negative emotions they may have experienced in the last week. The ten positive emotions were *Admiration, Calm, Compassion, Determination, Feeling moved, Gratitude, Hope, Love, Relief, and Sensory pleasure*. The ten negative emotions were *Anger, Anxiety/Worry, Boredom, Confusion, Disgust, Fear, Frustration, Loneliness, Regret, and Sadness*. All ratings were made on a 7-point rating scale, ranging between 0 (not at all) and 6 (very much). Following the approach of Carstensen et al. (2020), the measure of *Positive emotions* was a composite rating of the ten positive emotions (UK sample: *M* = 3.46, *SD* = 0.51, Cronbach’s *α* = 0.85; US sample: *M* = 3.32, *SD* = 0.62, Cronbach’s *α* = 0.87); and the measure of *Negative emotions* was a composite measure of the ten negative feelings (UK sample: *M* = 2.60, *SD* = 0.57, Cronbach’s *α* = 0.88; US sample: *M* = 2.16, *SD* = 0.55, Cronbach’s *α* = 0.90).

#### Resilience

A total of four questions were partially composed to assessed participants’ level of resilience. Based on a validated scale to measure resilience (e.g., the *Brief Resilience Scale;* Smith et al., 2008), two items were composed to measure resilience self-efficacy (e.g., “*I feel that in very difficult situations I am able to respond in positive ways*”; “*I have a high capacity to overcome setbacks*”; Not at all = 0; Very much = 6). In addition, based on a validated scale of positive adaptation (e.g., *Antonovsky’s sense of coherence scale*; Eriksson and Lindström, 2005), two questions were composted to measure flourishing (e.g., “*I have the feeling that I lead a purposeful and meaningful life*”; “*I feel good about myself*”; Not at all = 0; Very much = 6). The mean score of Resilience for the UK sample was 3.66 (*SD* = 0.26; Cronbach’s *α* = 0.89) and for the US sample was 3.97 (*SD* = 0.14; Cronbach’s *α* = 0.90).

### Procedure

The two measures (1) emotional experiences and (2) resilience were presented to the participants in random order. Of note, the survey also addressed a range of other topics related to the impact of the COVID-19 on participants’ feelings, perceptions, and behaviors (fully described in Sun et al., 2020). Here, we utilized a subset of these variables directly related to the current research question. Furthermore, while Sun et al., (2020) were interested in the role particular emotions may have on multiple facets of wellbeing, the current study operationalized the measurements of global levels of either positive or negative emotions (i.e., scores on all relevant items were averaged together to create measurements of positive and negative emotions), and their relationships to resilience were explored. Thus, all the analyses reported below are original and have not been previously published elsewhere.

## Results

### Preliminary Analysis

#### Emotional Experience

First, we investigated the relative presence of positive vs. negative emotions and their interrelationship. Findings showed that overall, participants experienced higher levels of positive emotions than negative emotions (for the UK sample: *t*(999) = 15.4, *p* < 0.001, Cohen’s *d* = 0.486; for the US sample: *t*(996) = 18.7, *p* < 0.001, Cohen’s *d* = 0.593). In addition, there was a small but significant inverse relationship between positive and negative emotions (for the UK sample: *r* = −0.11, *p* < 0.001; for the US sample: *r* = −0.24, *p* < 0.001). This suggests that individuals who report more positive emotions experienced, in general, fewer negative emotions. However, in the current study, positive and negative emotions had a small overlap (estimated as 1–4%, based on *r*^2^ values), allowing us to investigate their unique contributions to resilience.

### Main Analysis

#### Resilience

To test whether individuals’ levels of positive and negative emotions were associated with their resilient mindset during the COVID-19 pandemic, we conducted a multiple regression analysis separately for each sample (UK and US). Positive emotions (PE), negative emotions (NE), and their interaction were entered as predictors, and resilience was entered as the dependent variable, in each one of the analyses. In the first step, we entered into the model PE and NE (standardized) variables, while in the second step, their interaction component was added. The significance of all effects was assessed using a bootstrap technique with 5,000 samples to overcome normality violations. The two models were significant and explained 48–49% of the variance in resilience: UK: *F*(3, 996) = 321, *p* < 0.001; US: *F*(3, 993) = 301, *p* < 0.001 (see Table 1 for all statistics). Adding the interaction component in the second step significantly (albeit weakly) increased the explained variance in both models: UK: *F*(1, 996) = 10.10, *p* = 0.002, $\Delta R_{\mathrm{adj}}^{2}$ = 0.5%; US: *F*(1, 993) = 10.20, *p* < 0.001, $\Delta R_{\mathrm{adj}}^{2}$ = 0.5%. The variance in resilience explained by PE alone ranged from 23 to 27%; the variance in resilience explained by NE alone was 17%; and the variance in resilience explained by the interaction between PE and NE was 0.5% [explained variance calculated based on beta-squared values (*β*^2^), detailed in Table 1].

**Table 1:** Standardized weights of positive emotions, negative emotions, and their interaction component, in accounting for individual differences in cultivating a resilient mindset during the COVID-19 pandemic, using separate linear hierarchical regression analyses for the UK and the US samples (*N*_total_ = 2,000).

Sample	UK (*N* = 1,000)		US (*N* = 1,000)	
	*β* [95% CI]	$R_{adj}^{2}$	*β* [95% CI]	$R_{adj}^{2}$
*Step 1*		0.486		0.470
Positive emotions	0.513^***^ [0.468, 0.558]		0.467^***^ [0.420, 0.513]	
Negative emotions	−0.419^***^ [−0.464, −0.374]		−0.410^***^ [−0.456, −0.363]	
*Step 2*		0.490		0.475
Positive emotions	0.516^***^ [0.471, 0.560]		0.480^***^ [0.433, 0.526]	
Negative emotions	−0.415^***^ [−0.460, −0.370]		−0.409^***^ [−0.455, −0.362]	
Interaction (PE, NE)	0.065^**^ [0.025, 0.105]		0.070^***^ [0.028, 0.119]	

** *p* < 0.01;

*** *p* < 0.001.

**Figure 1:**
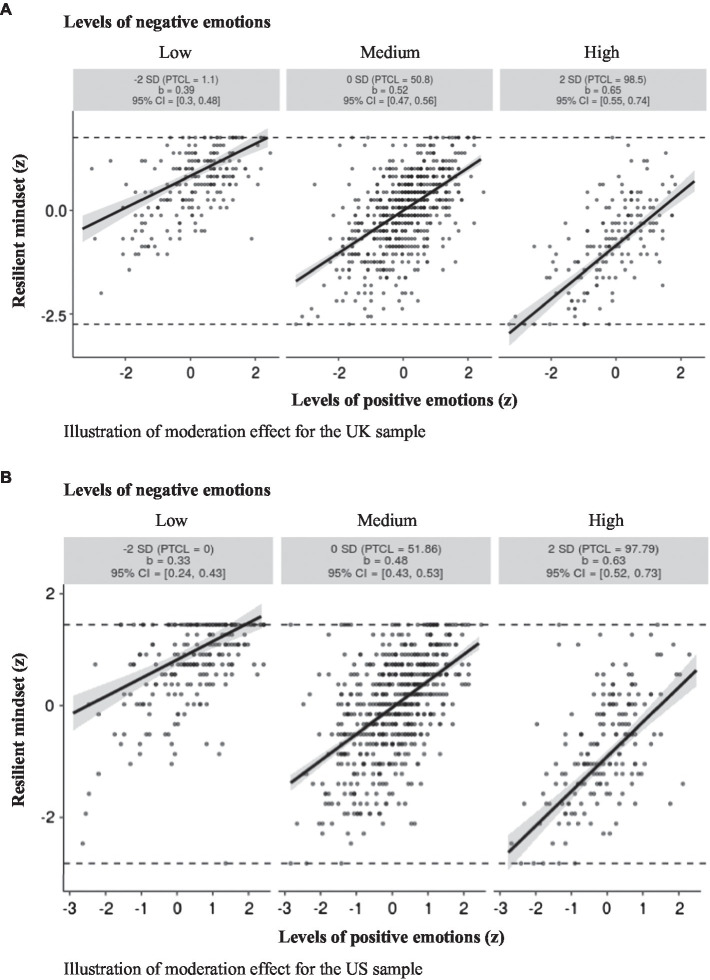
Resilient mindset (standardized) as a function of an individual’s level of positive emotions (standardized), illustrated for very high (+2SD above mean), average (at the mean), and very low (−2SD below mean) levels of negative emotions, for the UK sample **(A)** and for the US sample **(B)**. Each graphic shows the computed 95% confidence region (shaded area) and the full range of the observed data (gray circles). CI, confidence interval.

The results of the regression analyses indicate that the experience of positive emotions is positively associated with a resilient mindset and that the experience of negative emotions is negatively related to resilience. We also compared the effect size attributed to positive vs. negative emotions, using the procedure suggested by Diedenhofen and Musch (2015), and found positive emotions were significant, stronger predictors of resilience than negative emotions. This finding was robust across both samples: for UK: *Z* = 2.89, *p* = 004; for US: *Z* = 2.11, *p* = 036. Finally, in both samples, we also found a small but significant interaction indicating that the relationship between positive emotions and resilience is stronger for individuals who experience high levels of negative emotions than for those experiencing low negativity. Figure 1 illustrates these effects.

## Discussion

Around the world, people are experiencing a high degree of stress associated with the prolonged COVID-19 pandemic. In such challenging circumstances, the ability to confront a stressful reality is believed to depend on resilience. The current research found that the presence of positive vs. negative emotions differently relates to cultivating a resilient mindset. Individuals who experience high levels of positive emotions also report a higher level of resilience, whereas individuals who experience high levels of negative emotions show poorer resilience. These findings are consistent with existing research showing that positive emotions promote coping with acute stress encounters (e.g., Folkman, 1997; Fredrickson et al., 2003; Moskowitz et al., 2020). Thus, the current study expands the scope of the existing knowledge by suggesting that this premise is also relevant in the context of prolonged stress encounters, such as the current global crisis (for a similar conclusion, see Sun et al., 2020). Furthermore, the current results point to differences in how positive vs. negative emotions are related to resilience. Specifically, the findings show that positive emotions are associated with enhanced resilience to a greater extent than the deteriorated resilience associated with negative emotions. Hence, it can be suggested that boosting positive emotions may serve as a better channel than pursuing the elimination of negative emotions, for supporting people to live with their existing sorrow and feelings of loss.

In addition, in both samples, the overall level of positive emotions was higher than the level of negative emotions. This may lead to the proposition that the dominant effect of positive emotions on resilience exists only for individuals who experience fewer negative (compared to positive) emotions (see Huppert, 2009). However, contrary to this suggestion, a small but significant interaction effect was found in both samples, indicating that the relationship between positive emotions and resilience is *more* substantial for individuals who experience *high* levels of negative emotions. This result goes beyond the scope of the existing knowledge on resilience, as one could expect precisely the opposite – that the effect of positive experiences will diminish in a context of heightened stress, which is linked to deteriorated wellbeing (see Shigemura et al., 2020). Nonetheless, the current findings suggest an enhanced protective role of positive emotions that becomes even more pronounced for individuals or during circumstances that involve intensified levels of negative emotions, such as the current prolonged pandemic. This finding fits well with the existential PP2.0 perspective, claiming that existential wishes for meaning and love balance the common feelings of shame and fear, and that even negative emotions can be beneficial to wellbeing when they are transformed or overcome (Wong, 2011, 2019). It also supports the notion that resilience is a central mechanism of self-transcendence (e.g., Van Tongeren and Showalter Van Tongeren, 2020; Wong, 2020). Namely, the current findings suggest that positive emotions play a pivotal role in cultivating a resilient mindset, and point to the potential of positive emotions to transform suffering and thereby ameliorate the negative impact of the present collective crisis.

## Limitation

Three potential limitations should be noted. First, the study used a correlational design to assess the relationship between positive vs. negative emotions and resilience. Experimental designs are required to establish causality by examining whether momentary emotional experiences predict subsequent resilience levels. Second, the measurements of emotions and resilience were assessed using self-reports, which may not accurately reflect participants’ actual emotional status or resilience level. Future research could experimentally manipulate the feelings of positive and negative emotions, rather than assess them using self-report. Similarly, resilience can be measured using more objective behavioral indices. Although these paradigms will unavoidably lower sample size and thus limit the generalization of findings, they could clarify the exact role of positive and negative emotional experiences plays in cultivating resilience. Third, the current study measured resilience while focusing on facets of self-efficacy and flourishing, which are a subset of factors contributing to psychological resilience. The ideal operationalization of resilience should be based on longitudinal assessments of individuals’ level successful adaptation to stressors in their life (Kalisch et al., 2017). Future studies that will utilize longitudinal (and more comprehensive) assessments of psychological resilience will shed important light on the dynamic process of resilience.

## Conclusion

In two large, representative, and independent samples, this study examined the relative role of positive vs. negative emotions in accounting for individual differences in resilience in the context of high-stress situations, associated with the COVID-19 pandemic. The findings suggest that positive emotions are strongly linked with resilience in times of prolonged stress and that this effect is particularly evident among individuals who experience more (as opposed to less) negative emotions alongside their positive emotions. Thus, while suffering may be an inevitable part of human existence, it is not a situation to be avoided; rather, negative feelings (that can be labeled as suffering) are also the key to flourishing. They can make a unique contribution to wellbeing once they are transformed. This suggests that the main challenge to human beings is the pursuit of balancing aversive feelings with positive ones.

## Data Availability Statement

The datasets generated for this study are available on request to the corresponding author.

## Ethics Statement

The studies involving human participants were reviewed and approved by The Ethics Committee of the Psychology Department at the University of Amsterdam approved the study. The patients/participants provided their written informed consent to participate in this study.

## Author Contributions

The author confirms being the sole contributor of this work and has approved it for publication.

### Conflict of Interest

The author declares that the research was conducted in the absence of any commercial or financial relationships that could be construed as a potential conflict of interest.
